# Supplementary material for: A Study of Soil-Borne Fusarium Wilt in Continuous Cropping Chrysanthemum Cultivar ‘Guangyu’ in Henan, China
Source: J Fungi (Basel). 2023 Dec 27;10(1):14. doi: 10.3390/jof10010014 (PMC10820174; doi:10.3390/jof10010014)
Supplement: Supplementary file 1 [file jof-10-00014-s001.zip › jof-2742287-supplementary.pdf]

A study of soil-borne Fusarium wilt in continuous cropping Chrysanthemum cultivar 'Guangyu' in Henan, China

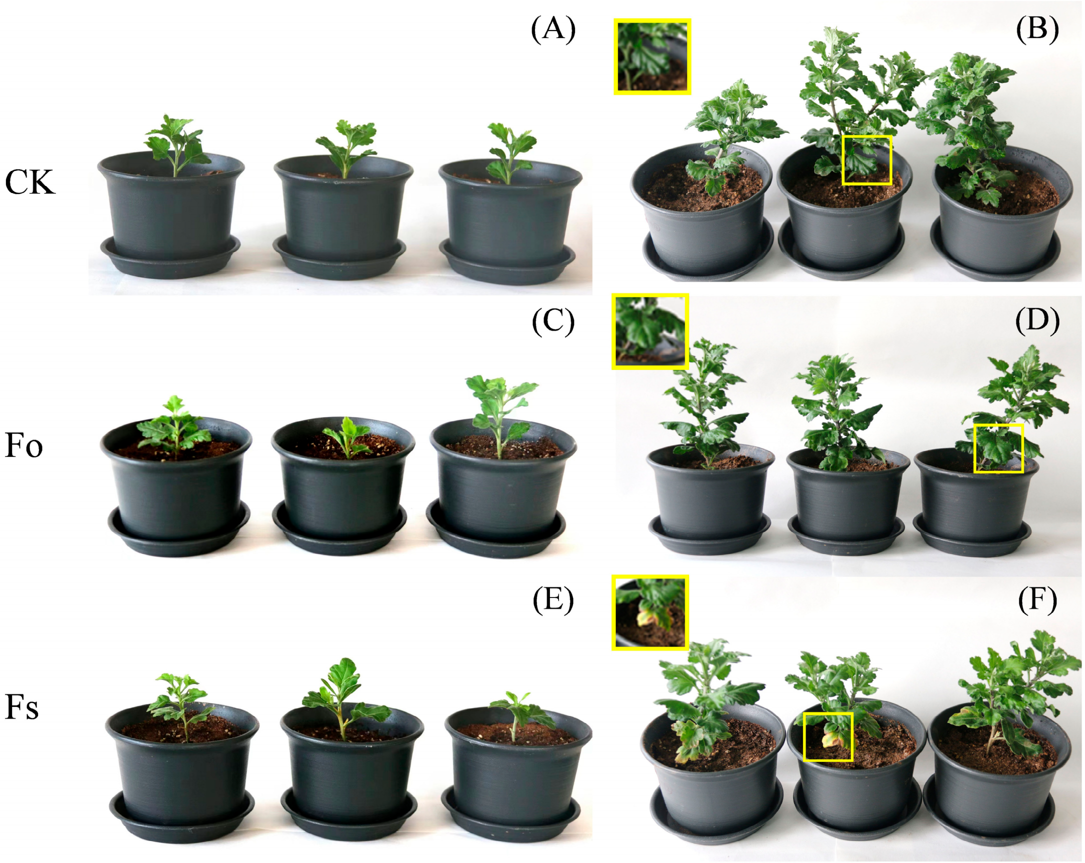

Figure S1. Pathogenicity test of Fo and Fs. (A, C, and E) Symptoms of each treatment group at 0 dpi. (B, D, and F) Symptoms of each treatment group at 30 dpi.

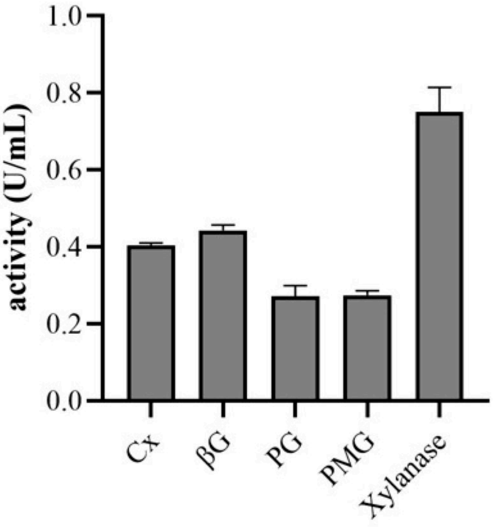

Figure S2. Activity of cell-wall degrading enzymes produced by *F. solani*: cellulase (CX), β-glucosidase (βG), pectin methylgalactuionase (PMG), and polygalacturonase (PG).

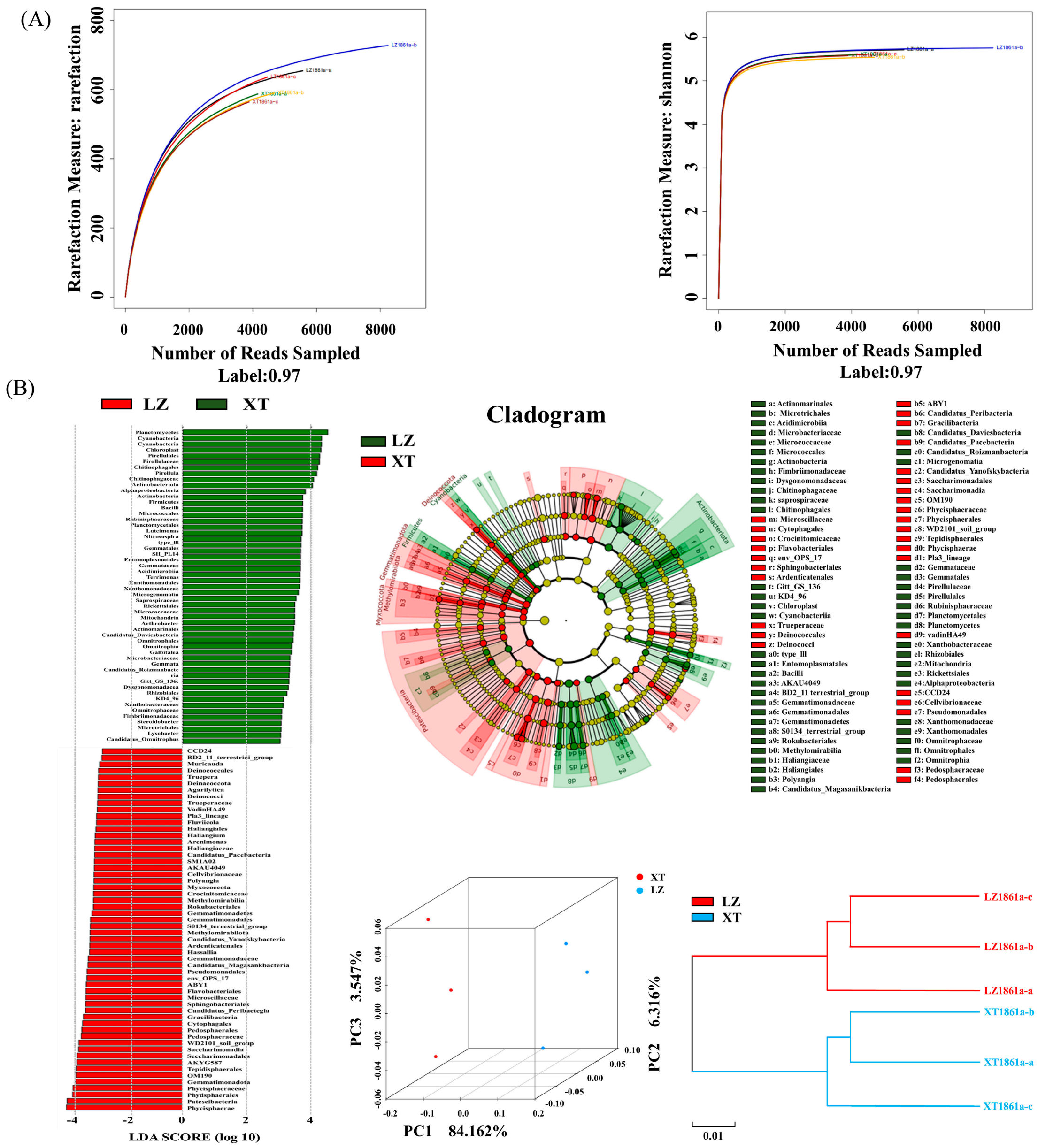

Figure S3. Analysis of soil bacterial microbial community structure: (A) sample sparsity curve and Shannon index. (B) Sample difference analysis, LEfSE analysis, PCoA principal coordinate analysis, and sample similarity tree.

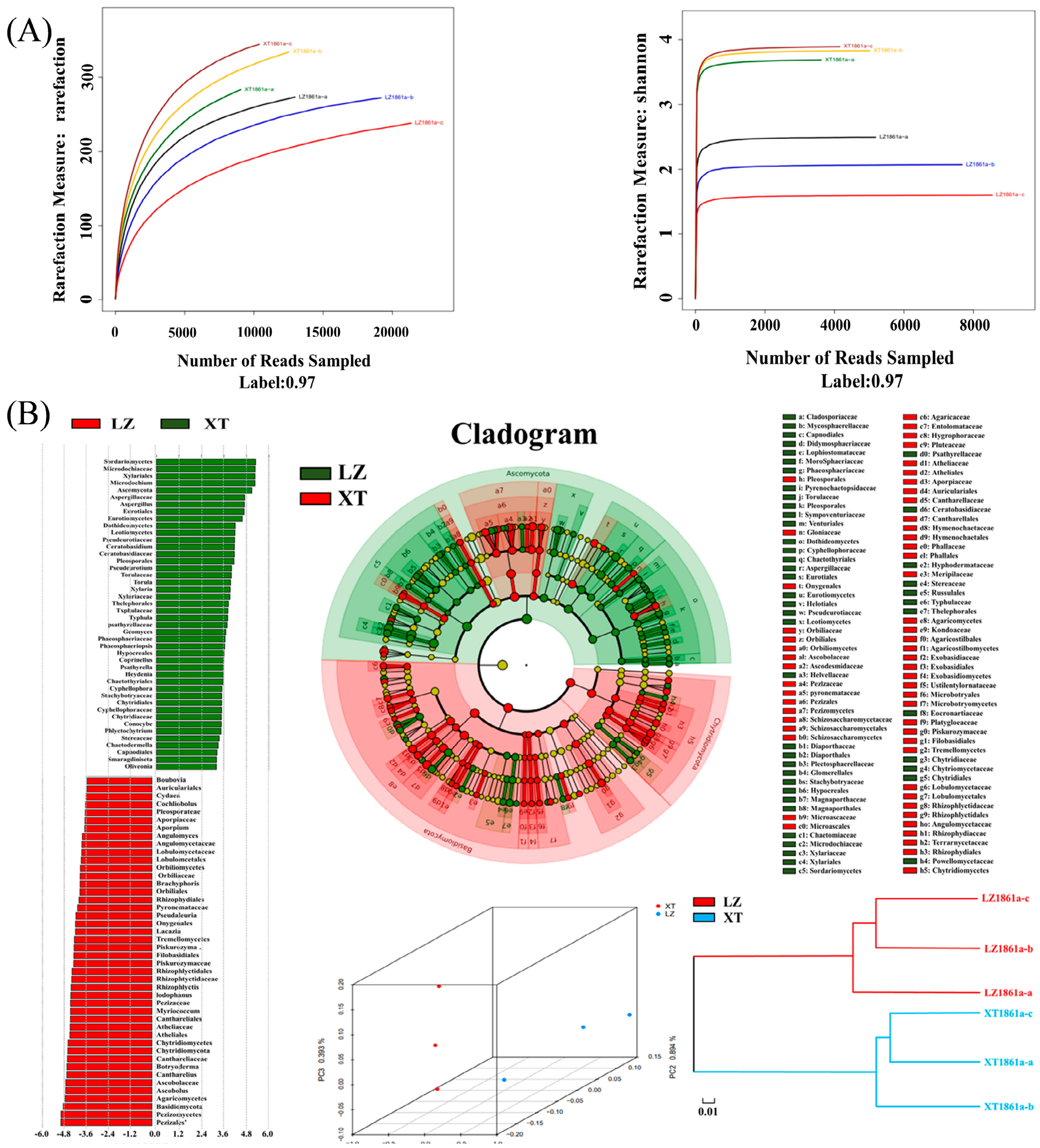

Figure S4. Analysis of soil fungal microbial community structure: (A)Sample sparsity curve and Shannon index. (B) Sample difference analysis, LEfSE analysis, PCoA principal coordinate analysis, and sample similarity tree.

Table S1 Statistics of disease incidence and index on 10th and 20th days

	10 d		20 d	
	**Incidence（%）**	**Index（%）**	**Incidence（%）**	**Index（%）**
CK	0	0	0	0
Fs	53.85	42.31	92.30	65.38

Table S2 Bacterial sequencing information and α diversity of soil samples

Sample	Reads	Chao	Richness	Shannon	Simpson	ACE	Evenness	coverage
XT1861a-a	4155	698.839	587	8.074	0.0086	681.063	0.877876	0.966306
XT1861a-b	4684	656.6105	590	7.9932	0.0095	655.8923	0.868405	0.975875
XT1861a-c	3881	655.1413	564	8.045	0.0088	651.1353	0.880247	0.966503
LZ1861a-a	5567	699	654	8.2452	0.009	704.4311	0.881547	0.982037
LZ1861a-b	8245	790.4615	727	8.3026	0.0084	773.3701	0.873427	0.987871
LZ1861a-c	4454	727.7179	636	8.0895	0.0105	734.0979	0.868635	0.966996

Table S3 Fungus sequencing information and α diversity of soil samples

Sample	Reads	Chao	Richness	Shannon	Simpson	ACE	Evenness	coverage
XT1861a-a	9045	354	283	5.316	0.0667	339.8444	0.652702	0.99215
XT1861a-b	12540	387.6666	334	5.5238	0.059	386.8033	0.658879	0.994418
XT1861a-c	10391	375.1886	344	5.6114	0.0707	378.7985	0.665953	0.994418
LZ1861a-a	12960	304.3333	273	3.5954	0.3412	303.4136	0.444278	0.996296
LZ1861a-b	19181	303.875	272	2.9845	0.4297	308.2603	0.369036	0.997341
LZ1861a-c	21351	299	238	2.3081	0.5428	292.507	0.292361	0.997143
